# Linking landscape-scale conservation to regional and continental outcomes for a migratory species

**DOI:** 10.1038/s41598-020-61058-3

**Published:** 2020-03-18

**Authors:** B. J. Mattsson, J. H. Devries, J. A. Dubovsky, D. Semmens, W. E. Thogmartin, J. J. Derbridge, L. Lopez-Hoffman

**Affiliations:** 1 Institute of Wildlife Biology and Game Management, University of Natural Resources and Life Sciences, Vienna, 1180 Austria; 2 Ducks Unlimited Canada, Stonewall, MB R0C2Z0 Canada; 3 Division of Migratory Bird Management, U.S. Fish and Wildlife Service, Lakewood, CO 80215 USA; 4 Geosciences and Environmental Change Science Center, U.S. Geological Survey, Denver, CO 80225 USA; 5 Upper Midwest Environmental Sciences Center, U.S. Geological Survey, La Crosse, WI 54603 USA; 6 School of Natural Resources and Environment, The University of Arizona, Tucson, AZ 85719 USA; 7 Udall Center for Studies in Public Policy, The University of Arizona, Tucson, AZ 85719 USA

**Keywords:** Animal migration, Conservation biology, Ecological modelling, Environmental economics, Population dynamics

## Abstract

Land-use intensification on arable land is expanding and posing a threat to biodiversity and ecosystem services worldwide. We develop methods to link funding for avian breeding habitat conservation and management at landscape scales to equilibrium abundance of a migratory species at the continental scale. We apply this novel approach to a harvested bird valued by birders and hunters in North America, the northern pintail duck (*Anas acuta*), a species well below its population goal. Based on empirical observations from 2007–2016, habitat conservation investments for waterfowl cost $313 M and affected <2% of the pintail’s primary breeding area in the Prairie Pothole Region of Canada. Realistic scenarios for harvest and habitat conservation costing an estimated $588 M (2016 USD) led to predicted pintail population sizes <3 M when assuming average parameter values. Accounting for parameter uncertainty, converting 70–100% of these croplands to idle grassland (cost: $35.7B–50B) is required to achieve the continental population goal of 4 M individuals under the current harvest policy. Using our work as a starting point, we propose continued development of modeling approaches that link conservation funding, habitat delivery, and population response to better integrate conservation efforts and harvest management of economically important migratory species.

## Introduction

The expansion of intensive agriculture threatens the diversity of species^1–4^ and ecosystem services^5^ associated with arable landscapes. Growing demands for food, feed, fuel, and fiber have precipitated such land-use intensification in these and other landscapes worldwide^6,7^. Substantial evidence shows that people benefit from high levels of biodiversity through many associated ecosystem services including food security, water quality, and recreation^8,9^. Birds are valued by the general public and recreationists, including birders and hunters in the case of game species^10–12^. Some grassland and farmland birds have undergone especially steep declines tied to intensifying agricultural practices^13^. Many of these populations comprise individuals undertaking annual long-distance movements between breeding and wintering grounds often spanning multiple countries^14,15^. International funds for conserving critical habitats at the landscape scale in North America are essential for maintaining these populations^16,17^ and these resources vary over time^18^. This conservation challenge begs the question of how shifts in funding and resultant changes in landscape composition might affect migratory birds.

Important advances have been made in modeling impacts of habitat changes on migratory birds at regional and continental scales. Existing approaches have important limitations, however. Some lack landscape-level scenarios for habitat conservation^19–21^, while others focus on hypothetical species^22^. Case studies most commonly lack predicted changes in species-specific abundance, predicting instead shifts in community-level metrics^23,24^ or indices of habitat suitability^25–27^. Species-specific predictions allow for evaluation of trade-offs and synergies regarding contrasting outcomes among species^28–30^. These issues become more complex when considering populations managed through both habitat conservation and harvest^31,32^.

Responding to this great challenge, methods and knowledge have been expanding to examine linkages between changing management and abundances of migratory birds at the continental scale. Seminal work on this topic emerged with a simple algebraic formulation for examining how abundance changes as a function of conditions on breeding and wintering grounds^33^. Later this theory was expanded to include density-dependent regulation of survival and reproduction when perturbing habitat during breeding and wintering periods^34^. More complex approaches built on these initial theories by employing individual-based modeling and optimal foraging rules to investigate population-level impacts of gains and losses of non-breeding habitat^20,35,36^. Recent studies have used increasingly diverse approaches to investigate outcomes of habitat perturbations, including migratory network models^37,38^ and integrated population models^39,40^. Although the diversity of modeling approaches has increased, still lacking is the ability to link changes in habitat at the landscape scale to population-level outcomes for migratory species. This step is necessary for understanding the practical implications of shifts in finances for conserving critical habitats of migratory species, which are often located in multiple countries.

Full-annual-cycle population-projection (FAC-PP) models offer a useful framework for studying such linkages and for conducting population viability analysis (PVA)^41^. They offer specification of density dependence^19,21^ and carry-over effects^42^ when making population-level predictions about the consequences of changing habitat conditions throughout the annual cycle of a migratory species. Deterministic FAC-PP models allow researchers to focus on shifts in central tendency and to identify key hypotheses to be tested with more intensive approaches that account for environmental and demographic stochasticity. Although these models require specification of vital rates and transition probabilities between age and habitat states^43^, they offer a compromise between data-hungry methods (e.g., integrated population models)^44^ and more simplistic methods (e.g., migratory network models)^38^. Existing FAC population models, however, lack mechanisms for linking shifts in funding for landscape-scale conservation to changes in continental-scale population dynamics.

**Figure 1:**
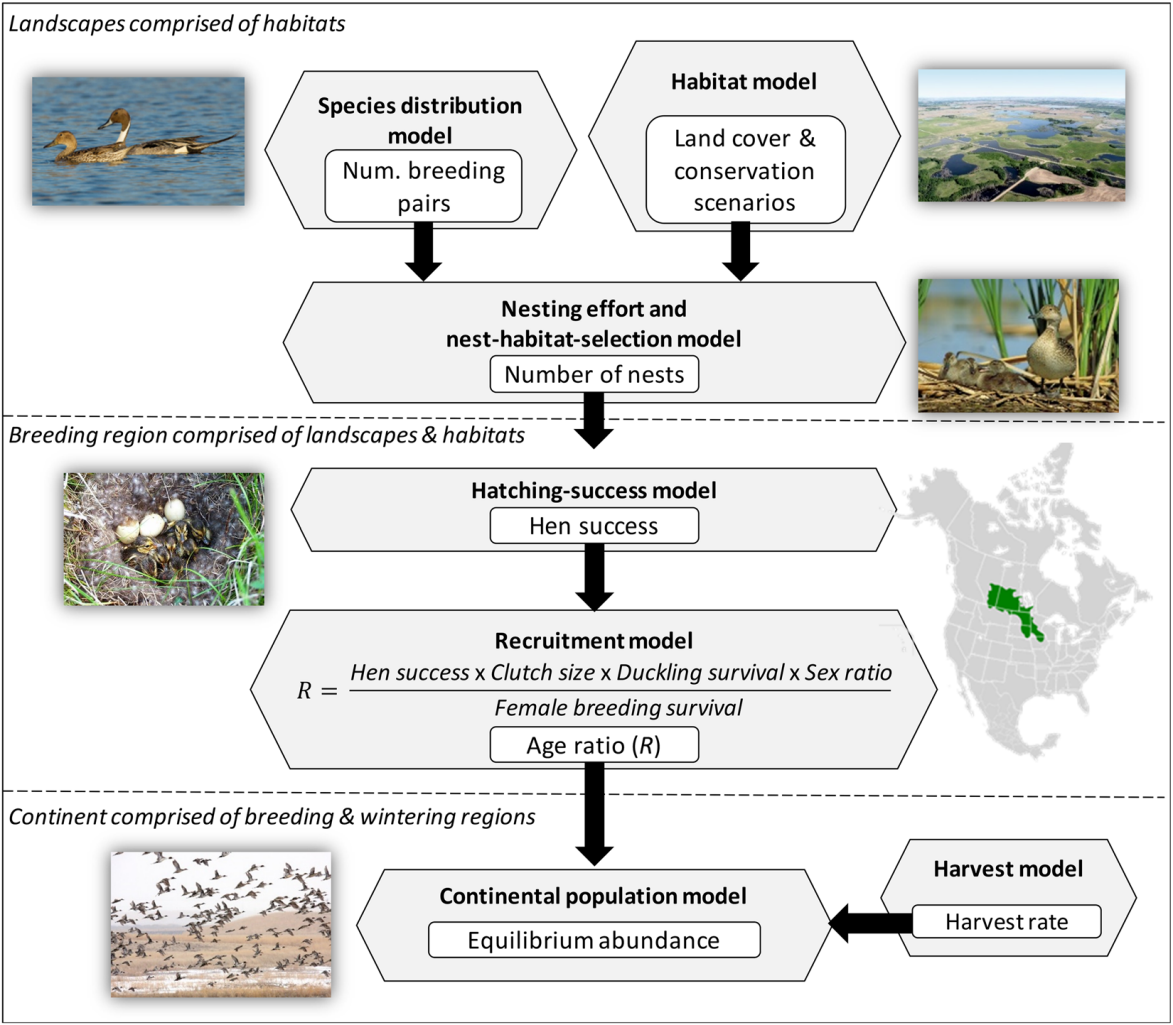
Modeling framework for estimating the impact of landscape-scale habitat conservation on continental-scale abundance of a migratory species while accounting for harvest. The framework links three landscape-scale, two regional-scale, and two continental-scale models. Gray hexagons represent submodels, and white boxes represent variables predicted at respective scales. Images were provided by ©Ducks Unlimited Canada under the Creative Commons Attribution 4.0 International Public License.

In this paper, we address the challenge of predicting population trajectories of a migratory bird at a continental scale under shifts in landscape-scale conservation within a core breeding region while accounting for hunting regulation across all regions. We develop a new modeling framework linking scenarios for funding and delivery of habitat conservation at landscape scales to continental-scale outcomes for the abundance of a migratory population. We do this through a spatially explicit model linking reproduction at a regional scale to population size at a continental scale, mediated by scenarios for habitat conservation and harvest at these respective scales (Fig. 1). The regional model predicts reproduction while accounting for breeding habitat availability and distribution of nests along with nest survival and renesting. Predicted reproduction then feeds into a full-annual-cycle population model for predicting abundance at the continental scale while accounting for hunting regulation across all regions.

We illustrate the utility of this framework using an empirical example of a migratory bird in North America, the northern pintail (*Anas acuta*; henceforth, pintail). Habitat conservation efforts for this species, and other upland-nesting waterfowl, focus on maintaining or restoring at-risk wetlands and grasslands along with promoting fall- versus spring-seeded crops to improve reproductive performance (i.e., nest survival) within the Prairie Pothole Region (PPR), a core breeding region for the species. We develop a priori scenarios representing varying landscape compositions as a function of funding for waterfowl habitat conservation in the PPR. We also explore post hoc scenarios to quantify percentages of spring-seeded cropland that would need to be converted into idle grassland to achieve particular population sizes at the continental scale. While the post hoc scenarios are likely to be unrealistic, we find it useful to explore the full theoretical range of habitat conversions. We also account for varying levels of hunting restrictions to better understand the joint consequences of harvest and habitat management on population dynamics.

Our starting scenario represents the estimated landscape-level habitat compositions during the focal study period (2007–2016) accounting for observed conservation investments during this timeframe (hereafter, Observed Conditions). We then generate alternative scenarios by modifying habitat compositions to represent the impact of plausible shifts in conservation investments (Table 1). The five alternative scenarios in order of increasing area conserved include: (1) removing conservation investments (No Conservation), (2) removing rotation of fall-seeded crops (No Winter Wheat), (3) maximizing rotation of fall-seeded crops in areas of high pintail density (Increase Winter Wheat - Targeted), (4) doubling conservation investment (Increase Conservation), and (5) maximizing rotation of fall-seeded crops across all landscapes (Increase Winter Wheat). The first two scenarios are expected to reduce continental population size by decreasing beneficial land cover, whereas the latter three scenarios are expected to increase continental population size. We also examine scenarios with and without annual harvest of pintails. We incorporate uncertainty by examining consequences of scenarios across a range of plausible parameter values for variables with existing empirical data, including reproduction, male breeding survival, and harvest rate. Although models at each scale have been useful on their own to study migratory-species conservation, combining these modeling frameworks while accounting for uncertainty enables novel insights about multi-scale responses to shifts in conservation funding.

**Table 1:** Scenarios representing observed and alternative levels of conservation funding and corresponding levels of protection and enhancement of habitat for northern pintails and other waterfowl within the Canadian Prairies 2007–2016. Costs are given in 2016 USD.

Scenario^a^	Conservation actions	Amount conserved		Cost ($M)^d^
		Area (km^2^)^b^	Pct.^c^	
Observed Conditions	Conversion of spring-seeded cropland to hayland/pasture	2,994	0.52	34
	Conversion of spring-seeded cropland to idle grassland	380	0.07	75
	Winter wheat included in cropland rotation	2,724	0.47	19
	Protection of existing grasslands	3,910	0.68	105
	Protection of existing wetlands	1,146	0.2	64
	Restoration of wetlands	59	0.01	17
	*Subtotal*	*11,213*	*1.95*	313
**Habitat Loss**				
No Conservation	Conservation removed	0	0	0
No Winter Wheat	Observed Conditions	11,213	1.95	313
	Winter wheat excluded from cropland rotation	−2,724	−0.47	−19
	*Subtotal*	*8,489*	*1.48*	294
**Additional Conservation**				
Increase Winter Wheat - Targeted	No Winter Wheat scenario	8,489	1.48	294
	Winter wheat included in cropland rotation	2,109	0.37	15
	*Subtotal*	*10,598*	*1.85*	309
Increase Conservation	Conversion of spring-seeded cropland to hayland/pasture	5,987	1.04	68
	Conversion of spring-seeded cropland to idle grassland	759	0.13	150
	Winter wheat included in cropland rotation	2,724	0.47	19
	Protection of existing grasslands	7,820	1.36	210
	Protection of existing wetlands	2,293	0.4	127
	Restoration of wetlands	119	0.02	33
	*Subtotal*	*19,702*	*3.43*	588
Increase Winter Wheat	No Winter Wheat scenario	8,489	1.48	294
	Winter wheat included in cropland rotation	23,130	4.03	160
	*Subtotal*	*31,619*	*5.51*	454

^a^Scenarios represent estimated 2016 landscape compositions under (a) Observed Conditions: 2007–2016 conservation accomplishments intact, (b) No Conservation: 2007–2016 conservation accomplishments removed, (c) No Winter Wheat: removal of winter wheat from Observed Conditions, (d) Increase Winter Wheat - Targeted: maximize winter wheat only in landscapes with >2.3 pintail pairs/km^2^, (e) Increase Conservation: double the 2007–2016 conservation accomplishments (except winter wheat), and f) Increase Winter Wheat: maximize winter wheat (to 30% of spring-seeded wheat).

^b^Acreage added across Canadian Prairies as of 2016 relative to No Conservation.

^c^Percent of total area in the Canadian Prairies.

^d^Cumulative cost of habitat conservation over the 10-year period.

## Results

### Distribution of nests

The distribution of nests varied little among scenarios, and nests were distributed according to habitat availability (Table 2). The largest proportion of nests occurred in spring-seeded cropland followed by grazed grassland > hayland = wetland > idle grassland > fall-seeded croplands including winter wheat. Pairwise differences between scenarios, with respect to proportions of nests by habitat type, ranged from 0% to 6%. The largest contrast was in the percentage of nests within spring-seeded cropland, which decreased from 51% to 44% when comparing No Winter Wheat to Increase Winter Wheat. The distribution of nests under Observed Conditions was most similar to that under No Winter Wheat and Increase Winter Wheat - Targeted, with <1% difference.

**Table 2:** Expected distribution of pintail nests under alternative conservation scenarios within landscapes of the Prairie Pothole Region of North America 2007–2016.

Scenario	Proportion of nests by land-cover type					
	Spring-seeded cropland	Fall-seeded cropland	Grazed grassland	Idle grassland	Hayland	Wetland
No Conservation	0.508	0.001	0.041	0.293	0.079	0.079
Observed Conditions	0.485	0.006	0.040	0.308	0.081	0.080
Increase Conservation	0.464	0.006	0.040	0.325	0.083	0.082
No Winter Wheat	0.490	0.001	0.040	0.308	0.081	0.080
Increase Winter Wheat	0.444	0.048	0.040	0.309	0.080	0.079
Increase Winter Wheat - Targeted	0.476	0.016	0.040	0.308	0.081	0.080

Distribution of nests was modeled as a function of pintail population distribution, pintail nest habitat preference, and altered habitat availability at the landscape scale.

### Reproduction

Under Observed Conditions, average hen success (i.e., number of hatched nests per adult female) was 0.189 (standard error [SE] = 0.016), and age ratio (i.e. the number of fledged females per adult female) was 0.471 (SE = 0.045). Average hen success ranged from 0.176 (SE = 0.014) under No Conservation to 0.204 (SE = 0.017) under Increase Conservation (Table 3), with a difference of 0.028 between these averages. Likewise for these scenarios, age ratio ranged from 0.440 (SE = 0.042) to 0.509 (SE = 0.049), with a difference of 0.069.

**Table 3:** Regional-scale reproduction and continental-scale equilibrium abundance (in millions) of northern pintails modeled under alternative scenarios for habitat conservation in the Prairie Pothole Region of North America.

Scenario	Hen success		Age ratio			Population size without harvest	
	Avg.	SE	Avg.	SE	95% Prediction interval	Avg.	95% Prediction interval
No Conservation	0.176	0.014	0.440	0.042	0.357–0.523	1.773	0.054–3.478
Observed Conditions	0.189	0.016	0.471	0.045	0.382–0.559	2.259	0.119–3.708
Increase Conservation	0.204	0.017	0.509	0.049	0.413–0.604	2.707	0.291–3.968
No Winter Wheat	0.187	0.015	0.467	0.045	0.379–0.555	2.213	0.110–3.684
Increase Winter Wheat	0.200	0.017	0.500	0.048	0.406–0.594	2.617	0.239–3.911
Increase Winter Wheat - Targeted	0.191	0.016	0.477	0.046	0.388–0.567	2.350	0.141–3.755

### Harvest rate

Assuming a daily bag limit of one pintail, a common and restrictive policy for this species^32,45^, percent of all individuals harvested (henceforth, harvest rate) averaged 9% (95% confidence interval [CI]: 5–15%) out of all pintails alive in the fall (i.e., pooled across age and sex cohorts). This corresponds with an average baseline harvest rate for adult females of 6% (95% CI: 3–10%).

### A priori scenarios

**Figure 2:**
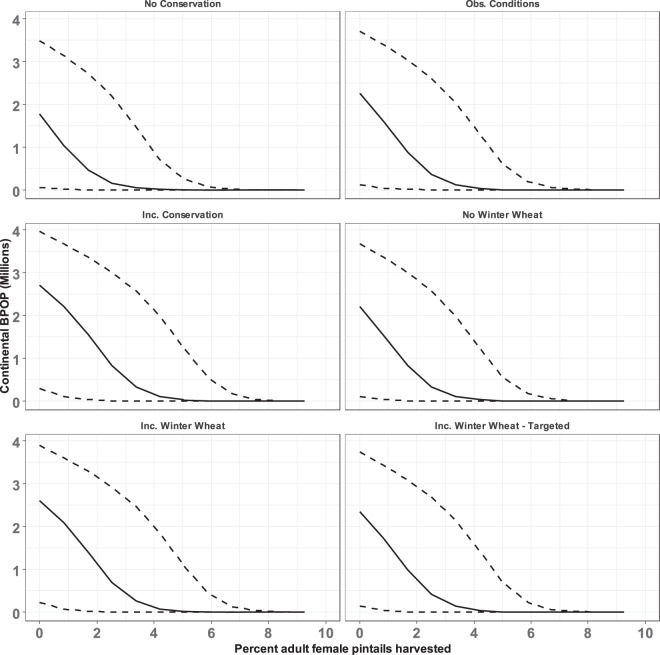
Predicted equilibrium population size at the start of breeding (BPOP) for northern pintails in North America as a function of baseline harvest rate under six a priori scenarios for habitat conservation in the Prairie Pothole Region of North America. Solid line is based on mean values for all parameters, and the dashed lines are based on the upper and lower 95% confidence intervals for parameters derived from available empirical data. Inc. = increase.

Under all a priori scenarios for habitat conservation (regardless of harvest) and average parameter values, the predicted number of male and female pintails at the start of breeding across all regions at equilibrium (henceforth, population) was predicted to reach <3 M individuals (Table 3 and Fig. 2). Under No Conservation and no harvest, we predicted a population of 1.8 M (95% CI: 54 K–3.5 M) pintails when using the lower 95% confidence limits (CLs) for the focal parameters. While still assuming average parameter values ≥5% baseline harvest led to extirpation across all of these scenarios, and 3% baseline harvest led to a population exceeding 1 M individuals under none of the scenarios except Increase Conservation (Fig. 2). The 10-year cost of this scenario was estimated at $588 M. The North American Waterfowl Management Plan (NAWMP) goal of 4 M pintails^46^ was reached under limited conditions: Increase Conservation scenario, no harvest, and upper 95% CLs of parameter values. With a bag limit of one pintail, this goal was not reached under these scenarios.

Across the a priori scenarios, habitat conservation affected 2 to 6% of the Canadian portion of the PPR (henceforth, Canadian Prairies; Table 1). Total cost of the observed conservation delivery (i.e., Observed Conditions scenario) during the 10-year study period was $313 M (Table 1). The remaining scenarios differed from Observed Conditions by >$10 M. The Increase Conservation scenario was the most expensive alternative: it would cost $275 M more than Observed Conditions and $134 M more than Increase Winter Wheat. Avoiding expenditures on winter wheat would cost $19 M less, and targeted rotations of winter wheat (Increase Winter Wheat - Targeted) would cost $15 M more than Observed Conditions. Cost per unit area differed substantially between types of habitat conservation delivered. Promoting winter wheat had the lowest cost ($69 ha^−1^), and wetland restoration had the highest cost ($2.8 K ha^−1^). The range of area conserved across scenarios other than No Conservation ranged from 8.5 K km^2^ to 32 K km^2^ across the extreme winter-wheat scenarios (i.e., No Winter Wheat and Increase Winter Wheat, respectively). Observed Conditions and Increase Winter Wheat - Targeted had intermediate areas conserved equal to 11 K km^2^. Under Observed Conservation, nearly 3000 km^2^ of spring-seeded cropland (ca. 1% of total) was converted to idle grassland at a cost of $75 M during the 10-year study period. We estimated a total of 250,200 km^2^ of spring-seeded cropland in the Canadian Prairies, with a one-time cost of $197,931 km^−2^ ($801 ac^−1^) when converting this cropland to idle grassland.

### Post hoc scenarios

**Figure 3:**
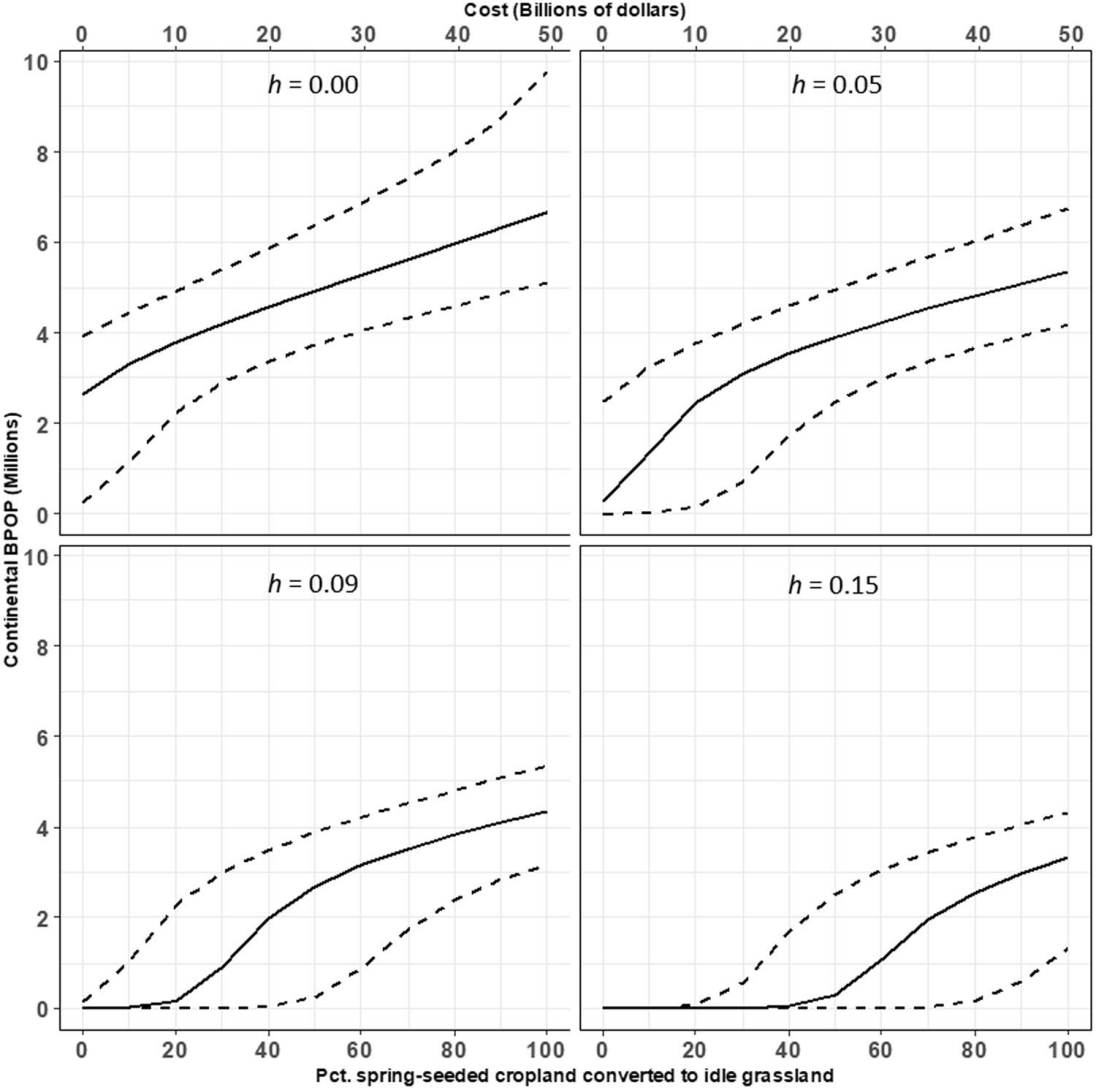
Predicted relationship between conversion of spring-seeded cropland to idle grassland within the Prairie Pothole Region and the equilibrium population size of northern pintails at the start of the breeding season. The population size at the x-intercept (i.e., % converted = 0) corresponds with the Increase Winter Wheat scenario. Panels represent predicted relationships under particular proportions of all pintails harvested (i.e., harvest rate; *h*). A harvest rate of zero represents a closed hunting season, and the remaining represent the uncertainty about harvest rate expected under a bag limit of one pintail. Cost of habitat conversion (secondary x-axis) is given in 2016 USD. Solid line is based on mean values for all parameters, and the dashed lines are based on the upper and lower 95% confidence intervals for parameters derived from available empirical data.

Achieving a population of >2 M pintails under the restricted bag limit would require $9.9B to convert 20% of spring-seeded cropland (50,040 km^2^) to idle grassland, assuming 23 K km^2^ of cropland includes winter wheat in the rotation and the lower CL for harvest rate along with averages for the other parameters (Fig. 3). Under the same conditions, half the spring-seeded cropland (125,100 km^2^) would need to be converted at a total cost of $24.8B to ensure that the lower 95% CL exceeds 2 M pintails. Reaching the NAWMP goal of 4 M pintails while assuming a bag limit of one pintail and average parameter values would require converting 90% of the spring-seeded cropland (225,180 km^2^) at a total cost of $44.6B. Converting all spring-seeded cropland to idle grassland at a cost of $49.5B while annually harvesting 5% of pintails (i.e., the lower 95% CL under restrictive harvest) would yield a population of 5.3 M (95% CI: 4.2 M, 6.7 M) while assuming average reproduction and male breeding survival.

### Uncertainty

Population estimates differed by −11 K to 25 K individuals and 0 to 18% when varying adult breeding survival between its lower and upper CLs (i.e., 0.89 and 0.99) across combinations of fixed values for age ratio (corresponding with habitat conservation scenarios in the PPR) and harvest rate (Fig. S1). Combinations of absolute and proportional differences in the population estimates were maximized when age ratio was lowest (i.e., No Conservation) and harvest rate was 1–3%. For example, when harvest was 2% under the No Conservation scenario, the absolute difference was 25 K and the proportional difference was 5%.

By contrast, population estimates differed by 0 to 4.6 M individuals and 47 to 200% when varying age ratio in the PPR between its lower and upper CLs (Table 3) across values for mean age ratio and harvest rate (Fig. S2). Here, the absolute and proportional differences in the population estimates reached their maxima when mean age ratio was lowest (i.e., No Conservation) with no harvest.

## Discussion

Tools are needed to quantify and understand impacts from the expansion of intensive agriculture on migratory species, especially in agriculturally dominated regions. Likewise, methods are needed to account for the influence of conservation and harvest management on populations of economic or cultural importance. We have developed a novel modeling framework for linking landscape-scale habitat conservation with continental population dynamics of a migratory bird. In our case study, we found that existing conservation practices and realistic alternatives appear to be insufficient to maintain population viability of this species under a restrictive hunting-bag limit. Based on model predictions accounting for parameter uncertainty, enormous increases in habitat conservation within the focal region and possibly eliminating harvest of the species would be necessary to allow for achieving population goals at the continental scale. Increased agricultural intensification, driven by the conversion of ca. 9 M hectares of idled cropland (summer fallow) to continuous spring-seeded cropland under minimum tillage, has occurred over the past three decades^47,48^. The resultant increase in availability and use of spring-seeded versus idle cropland for nesting is the likely driver of declining reproduction levels^49,50^. At the observed level of annual funding for waterfowl habitat conservation ($31.3 M yr^−1^), it would take >100 years to cover the cost of habitat conversion ($35.7B) needed to achieve a predicted population size meeting the NAWMP goal of 4 M pintails. This assessment assumes average parameter values and that future conservation activities are focused on the pintail^51,52^.

The predicted decline in continental-scale abundance of pintails under observed habitat conditions contradicts our previous findings using the same population model with regional age ratios based on expert elicitation^19^. We previously assumed that age ratios in breeding regions were negatively correlated with abundance due to density-dependent reproduction within each region. On further review we now believe that reproduction is only density-dependent at a continental scale, whereby a greater proportion of pintails settle in areas with lower reproduction as the spring population increases. This spring fly-over effect was part of the original population model^19^ and was retained in the current version. Thus, linking scales between landscape-level conservation and continental-level dynamics has provided new insights about the scale of density-dependent reproduction for this species.

In addition to our own modeling work based on a large dataset of individual nests and broods, evidence from PPR banding studies indicates that pintail reproduction has declined. For example, the trend in age ratio of pintails banded at the end of breeding in the Canadian Prairies decreased from 1.5 to 0.5 from 1960 to 2014 coincident with population decline^45^. Similar results have been found using such banding-based age ratios for regressing changes in reproduction with agricultural intensification in the Canadian Prairies^50^. Our estimates of reproductive rate based on long-term studies of nests, and assumptions regarding lack of density-dependent reproduction at regional scales, are therefore supported by models using independent data.

As with any multi-scale modeling framework, ours carries several assumptions about the accuracy of parameter values. There are several possible sources of bias, some of which are discussed elsewhere^19^, and it is beyond the scope of this study to address all of these. For predicting continental-scale abundance, we found that uncertainty about reproduction was three orders of magnitude more important than was uncertainty about male breeding survival. We therefore highlight two parameters with potential for inducing bias in the reproduction estimate. There are concerns that estimates of nest survival are biased low due to disturbance caused by human observers^53^, but this hypothesis was not supported for any guild of birds considered in a meta-analysis on this topic^54^. Further, there was no evidence of visitation effects on nest survival of radio-tagged female mallards in the Canadian Prairies^55^. We therefore expect that bias induced by nest observation was negligible in our study.

Second, we assumed the land-cover map provided an accurate assessment of the proportion of spring-seeded crops and grassland in the landscapes. Errors with remote sensing can induce biased estimates, such that percentage of crops in the landscape is underestimated and grassland is overestimated in the PPR^56^. If present, this bias would make pintail persistence even less likely than in our current predictions. In our previous work^19^, we identified several other sources of uncertainty for predicting continental-scale abundance of pintails as a function of management. Although we have addressed uncertainties about variables for which empirically based confidence intervals were estimable (i.e., harvest rate, reproduction, and male breeding survival), many remain to be examined.

Further, our estimated impact of conservation efforts on pintail reproduction is based on information collected in the Canadian Prairies. When modeling population dynamics at the continental scale, we assumed similar conservation efforts and effects on reproduction on both sides of the U.S.-Canada border bisecting the PPR. This assumption was necessary, as we were unable to acquire similar data for the U.S. portion. There is evidence suggesting that age ratio has responded similarly to agricultural intensification in the US portion as it does in the Canadian Prairies^50^. A critical need then is to obtain comparable information about habitat conservation and pintail reproduction within the U.S. portion, so that our predictions can be further validated.

When comparing the U.S. and Canadian portions of the PPR, another important difference is the trend in funding for conservation of waterfowl habitat. Based on our concurrent study on annual funding for conservation of waterfowl habitat in the PPR, finances in the Canadian Prairies were quite stable during the study period (range: $26 M–$46 M annually)^57^. By contrast in the U.S. portion, funding was greater and growing (range: $61–$163 M annually). We may have therefore underestimated reproduction in the PPR under Observed Conditions, considering that the U.S. portion may have conserved more habitat compared to the Canadian Prairies. Taken together, there are important contrasts between countries in documenting conserved habitats and consistency of funding for the conservation work. Future analyses in ecoregions straddling an international border must therefore account for international differences in conservation funding and resultant habitat management.

Despite calls to integrate the management of habitat and harvest under NAWMP^19,58^, optimal policies for harvesting waterfowl in North America are based on estimates of abundance and distribution as state variables rather than habitat metrics^59^. Our predictive framework provides the means necessary for this formal integration. Specifically, we link landscape-scale conditions of habitat conservation throughout the PPR to pintail reproduction and a continental-scale forecast for trend in population size. These elements are the necessary ingredients for formally integrating harvest and landscape-scale conservation to achieve population goals at the continental level. Until now, this essential link has been missing.

A precautionary approach to hunting regulations entails projecting population outcomes under alternative harvest policies while accounting for uncertainty, which is an important consideration in the adaptive harvest management of waterfowl^60,61^. The current harvest strategy for pintails focuses on uncertainty about the strength of density dependence for post-harvest survival^59^. Although addressing this uncertainty can improve predictions about continental dynamics^19^, we believe that uncertainty about the effect of habitat conservation on reproduction (along with the magnitude of the age ratio) is an equally if not more important concern. By considering alternate forms of density-dependent survival and of conservation-dependent reproduction, the key sources of uncertainty for dynamics of the pintail population can be addressed through adaptive management.

Explicitly accounting for costs of habitat conservation can help inform strategies to maintain or increase at-risk populations of migratory birds. Applying a cost constraint when investigating alternative strategies^62,63^ is a simple method to address limited funds for conserving migratory species. Accurate accounting for spatial variation in cost of habitat conservation was important for finding an optimal strategy that would save money while conserving breeding habitat for bobolinks (*Dolichonyx oryzivorus*)^64^. The bobolink study also revealed that cost determined the optimal mixture of habitat protection and restoration. Similarly, cost savings were revealed by a study that used management costs when optimizing conservation of stopover habitats of the pink-footed goose (*Anser brachyrhynchus*)^65^. Optimization with cost as a constraint, however, may not reveal a clear relationship between expenditures and population-level outcomes. Based on estimated costs in our study, we found that achieving the desired population size and harvest level at the continental scale would require multiple decades under the current level of investment. This timeline may be insufficient given the predicted decline in abundance.

New approaches are needed to overcome the challenge of conserving habitats for migratory birds in the face of limited budgets. The emerging field of biodiversity-based agriculture may hold some promise^66^. Methods in this field include model-based games^67^ that investigate scenarios for conserving both local and migratory biota. Our modeling approach can be used to support such investigations. Biodiversity-based agriculture requires advances in participatory methods for engaging stakeholders^22,68,69^ along with predictive models for species response^19,21^. Frameworks exist for integrating knowledge and stakeholder processes to inform decisions for conserving highly mobile species, including collaborative decision analysis^70^ and adaptive management^60,61^. These frameworks must be tested for improving the practice of conserving migratory species.

Across the annual cycle, migratory birds span multiple jurisdictions ranging over large extents. Linking landscape and continental scales is therefore needed when modeling population-level outcomes under realistic scenarios of habitat conservation. Such linkages are rarely modeled when investigating effects of habitat changes on migratory populations^71–73^. Modeling populations at extreme scales is challenging as it requires the integration of conceptual models and mathematical approaches along with diverse data sets^71,74^, which we have experienced in our work. This approach however can address important ecological questions in ways that were until now not possible.

Addressing scale mismatches between management and ecological processes is a critical factor limiting effective conservation^75^. Tools for developing multi-scale models and obtaining the necessary data are being increasingly refined and used^44^. A particular opportunity is extending our framework for other species and in novel contexts. Continental-scale population models and necessary data are now available for migratory species representing multiple taxonomic groups, including waterfowl^20,21^, passerines^76–79^, shorebirds^35,80^, butterflies^40^, and bats^38^. Landscape-level models are relatively common^69^, and so there is a great opportunity for linking landscape-level changes to continental-scale dynamics of migratory species. A future challenge for multi-scale modeling is forecasting annual changes in population size while accounting for shifts in weather and land cover induced by global change and nonstationarity^81,82^. Multi-scale modeling that links landscape-scale changes to continental-level outcomes offer an important means for closing the gap between the practices of management and science of conserving migratory species.

## Methods

### Study system for case study

**Figure 4:**
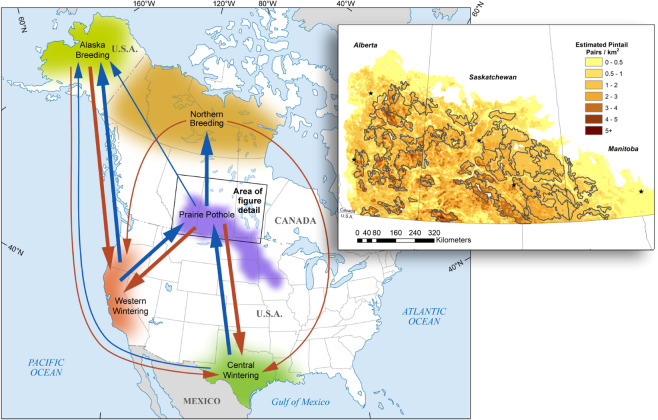
Fall (downward arrows) and spring (upward arrows) migration of the northern pintail between core breeding and wintering areas of North America. Arrow width scales with increasing proportion of pintails using each migratory pathway. The varying density of shading is a schematic to represent reduced pintail density along the periphery of each core region. Inset map shows long-term (1961–2009) average density of pintail pairs (pairs/km^2^) among conservation-planning target landscapes represented by gray outlines within the Canadian Prairies. Graphics are adapted from existing figures in the literature^43,49,51^.

The PPR of Canada and the northcentral U.S. comprises the formerly glaciated and wetland-rich region serving as the primary breeding area for many North American waterfowl^83–85^ (Fig. 4). Historically, extensive grasslands along with abundant and diverse wetlands provided ideal habitat for successful waterfowl reproduction in this region^86^. Since human settlement, however, a majority of the PPR has become an important agricultural production area for cereal grain, oil seed and row crops. This region is one of the most productive agricultural landscapes in the world^87^ with a high potential for expansion of cropland and agricultural intensification^4^. Conversion of grassland to annual cropland and drainage and degradation of wetlands have significantly altered the landscapes in which waterfowl settle to breed^88,89^. These habitat losses represent the primary conservation concern for NAWMP^90,91^.

Conversion of grasslands to cropland and associated alteration of predator communities in the PPR are thought to be the leading cause of long-term declines in waterfowl production in this region^88,92–94^. In addition, the intensity of cropping practices on existing cultivated lands has increased in recent decades. The largest and most economically and environmentally significant change in agricultural land use since the 1970s has been the decline in summer fallow. Through this practice, cropland was removed from production during alternate growing seasons for moisture accumulation, nitrogen release, and weed control^47^. In the Canadian Prairies, summer fallowing declined by approximately 90 K km^2^ from 1971 to 2016^95^. In its place, the prevailing approach is continuous cropping with minimum and zero-tillage practices (hereafter, ‘conservation tillage’) requiring high nutrient and pesticide inputs^47,48^. While beneficial for soil conservation, reduced stubble disturbance in combination with spring seeding operations may create ecological traps for breeding birds^96^.

Migratory waterfowl are one of the most intensively managed groups of vertebrates in North America. Ongoing restoration and protection of habitats throughout the annual cycle along with annual regulation of harvest rates comprise modern waterfowl management^60^. Every year, millions of dollars are invested in conservation efforts within the PPR^51,97,98^. Improving the effectiveness of waterfowl management depends on a better understanding of how changes in habitat as a result of conservation activity will affect achievement of continental-level goals for species abundance^19,58,99^.

Conservation programs for breeding waterfowl in the PPR are generally targeted toward habitat for dabbling ducks in general, not to any particular species^100^. Primary conservation activities in this region include conversion of cropland to forage grass (hay or pasture) and idle grass, protection of existing grassland and wetlands, restoration of drained wetlands, and converting spring-seeded cropland to fall-seeded varieties^51^. These habitat manipulations serve multiple objectives; they not only maintain or restore the carrying capacity of the landscape to attract and hold breeding ducks, but they also increase breeding success through improved nest survival^88,101,102^. These conservation investments are expected to provide benefits for migratory ducks over the long term, assuming recurring use of these habitats by target species^100,103^.

### Focal species

The pintail is of particular interest in its response to conservation actions. A significant population decline in the 1970s and 1980s caused concerns for hunters and bird enthusiasts, leading to its classification as a species of conservation concern^104^. Although the initial decline has been attributed to widespread prairie drought in the 1980’s, the leading hypothesis for lack of population recovery is agricultural intensification leading to reduced reproduction in the PPR^19^. Wetland loss, especially loss of small and shallow basins preferred by pintails, may have reduced the carrying capacity of the region to attract and hold pintail pairs^105,106^. Further, and likely more influential, have been changes in land use that have reduced the ability of pintails to successfully hatch nests^107–109^. Since 1990, the number of pintails at the start of breeding in North America has remained between 1.8 M and 3.6 M except for 2011 when the population was estimated at 4.5 M^110^.

Based on a population model for the PPR, declining productivity has been a main driver of changes in pintail abundance over the past five decades^50^. This study also found that productivity was negatively correlated with agricultural intensification at the subregional scale. Indeed a growing body of evidence from North America suggests that the decline in abundance of the pintail, a species that readily nests in residual crop stubble prior to seeding, may be linked to the increase in conservation tillage practices that reduce the availability of undisturbed stubble provided by fallowed cropland^49,50,108^. The economic value of birding and hunting associated with pintails in North America is estimated at >$100 M (2014 USD) annually^10^. A declining population is therefore not only a concern for biodiversity but also for society.

Unlike other dabbling ducks, pintails use croplands for nesting and begin nesting before crops are planted^102,104,111,112^. They therefore uniquely benefit from the high nest survival afforded by rotating crops between spring-seeded and fall-seeded crops such as winter wheat^102,111^. Pintails tend to settle opportunistically within the PPR in association with variably available wetlands, especially temporary and seasonal wetlands^113–115^. Predicting the impact of habitat conservation on the pintail population therefore requires an accounting of their distribution and habitat associations along with resultant changes in breeding success in affected landscapes. Such effects of amount and spatial targeting of conservation investments have until now not been investigated.

Modeling frameworks have been developed for predicting outcomes of habitat management for pintails at landscape to regional scales^49^ and regional to continental scales^19^. In addition, methods are emerging for explicitly quantifying the sources and patterns of funding for conservation of wildlife habitat in North America^57^. These developments render a great opportunity for linking modeling frameworks to predict the outcomes of funding scenarios for population dynamics of this migratory species.

Our study builds from previous work^19^ that used a FAC-PP model to investigate shifts in continental-scale population size and maximum sustainable yield caused by simulated changes in habitat quality within the PPR and within the Gulf Coast wintering region. The present study focuses on effects of landscape-scale habitat conservation within the PPR, as the previous study showed that increased age ratio in the PPR was more important than reducing the strength of density dependence for post-harvest survival in the Gulf Coast. The FAC modeling approach is needed to simultaneously account for effects of habitat conservation in the breeding grounds during summer and harvest across regions in the fall.

### Overview of modeling approach

Before giving a detailed description, we provide an overview of the modeling framework for linking landscape-scale conservation of habitat to continental dynamics of the pintail population. Our general approach was to relate landscape-level alterations to expected changes in the breeding population at equilibrium while accounting for parameter uncertainty. As such, we altered the observed landscape according to alternative conservation scenarios. More specifically, we updated a land-cover map to characterize the current composition of the landscape as it pertains to waterfowl in the PPR. We then used existing models for estimating pintail distribution, hen success, and ultimately age ratio throughout the PPR. Here, we accounted for uncertainty in multiple reproductive parameters. Next, we developed alternative scenarios for conservation of pintail habitat in the PPR along with an uncertain harvest rate of pintails at the continental scale. As a final step we adapted an existing approach for modeling population dynamics at the continental level as a function of varying age ratios, mediated by landscape-scale conservation, while accounting for harvest during fall across all core regions of North America. We ran the model three times for each scenario based on the mean and 95% confidence limits of parameters for which we could quantify uncertainty based on empirical data.

### Land-cover map and landscape-level habitat composition

We used Agriculture and Agri-Food Canada’s (AAFC) 2016 annual, 30-m crop raster (http://open.canada.ca/data/en/dataset/ba2645d5-4458-414d-b196-6303ac06c1c9) to estimate landscape composition in 2016 for eight habitat classes (spring-seeded cropland, fall-seeded cropland, idle grassland, grazed grassland, hayland, wetland, trees/shrubs, and other). Because wetlands are poorly captured in AAFC’s crop mapping layer, we recalculated base habitat composition after including estimated wetland habitat area from the CanVec hydrology layer^116^ after adding small wetlands missed by the AAFC and CanVec layers^49^. This approach yielded the 2016 land-cover map for modeling pintail distribution and reproduction throughout the PPR. To conduct the spatial analysis, we used ArcGIS version 10.4 (Environmental Systems Research Institute, Redlands, California, USA). Detailed methods for developing the land-cover map are provided elsewhere^49^.

From this layer, we estimated proportional habitat composition in each of 49 conservation-planning landscapes (henceforth, landscapes) covering 574 K km^2^ comprising the Canadian Prairies (Fig. 4)^51^. All except three of these are target landscapes that delineate areas of high breeding duck density. The landscapes range in size from 1.68 K–12.7 K km^2^ and collectively cover an area of 168 K km^2^. The remaining three landscapes, representing area outside target landscapes in each province, are much larger and range from 58.6 K–183 K km^2^.

### Reproduction model

To quantify the impact of conservation scenarios on pintail reproduction in the PPR, we estimated the age ratio (*R*) as the number of juvenile females divided by the number of adult females alive at the end of the breeding season. In particular, we estimated *R* using a reproduction model that was structured as follows:

1 $$R={S}_{h}\times C\times {S}_{d}\times \theta \times {S}_{f}^{-1}$$

where ${S}_{h}$ is proportion of hens having hatched a nest (i.e., hen success), *C* is mean clutch size at hatching, ${S}_{d}$ is the probability a duckling survives 30 days after hatching (i.e., to the end of the breeding season), $\theta$ is the proportion of hatchlings that are female, and ${S}_{f}$ is the probability that an adult female survives during the breeding season. This approach mimics that applied for estimating reproduction in previous avian population models^117^.

We estimated parameters for the reproduction model based on existing predictive models fit to empirical data from 992 nests that were monitored throughout the Canadian Prairies from 1993–2011^49,112,118^. Hen success (${S}_{h}$) was estimated as a function of habitat conditions. Specifically, we first accounted for the spatial variation in pintail breeding pair density within each landscape based on a species distribution model^49,118^ (Fig. 4) from which we extracted the estimated long-term average (1961–2009) abundance of breeding pairs in each landscape^51^. Estimated number of breeding pairs and proportional habitat availability in each landscape were used in an existing deterministic model of pintail nest habitat selection, habitat-specific nest survival, and breeding effort (nesting and renesting propensity) in the Canadian Prairies^49,112^ to estimate the number of hatched nests across the region. We divided hatched nests by the number of breeding pairs to estimate hen success (${S}_{h}$), which was then entered in Eq. 1 to estimate age ratio. Age ratio was subsequently used as an input parameter for the continental population model (see “Population model structure and parameters” below). As comparable information is missing from the U.S. portion of the PPR, we assumed that age ratios were equal on both sides of the border.

The remaining parameters for Eq. 1 were directly estimated from empirical data. Average clutch size (*C* = 8.0, SE = 0.05) was based on direct observations of 2925 nests monitored throughout the Canadian Prairies during 1993–2011 by Ducks Unlimited Canada (DUC; unpubl. data). Mean duckling survival (*S*_*d*_ = 0.505) was based on the average of annual estimates from pintails monitored with radio telemetry in southern Alberta during 1995 to 1996^119^. Consistent with other models of bird reproduction based on nesting studies^117^, we assumed that 50% of hatchlings are female. The survival rate for adult females (*S*_*f*_ = 0.81) during breeding was based on a telemetry study of pintails in an agricultural landscape within southern Saskatchewan during 1998–2000^120^. We estimated standard errors of duckling survival (0.020) and adult female breeding survival (0.016) based on respective coefficient of variation estimates from a large sample of radio-marked mallards in the Canadian Prairies from 27 sites during 1993–2000^103^.

### Scenarios for habitat conservation and pintail harvest

We used the reproduction model to explore the impact of scenarios representing alternate landscape compositions throughout conservation areas of the Canadian Prairies during 2007–2016 (Table 1). The 2016 land-cover map represented the Observed Conditions scenario of empirically derived habitat conditions resulting from investments in habitat conservation. Alternatives to Observed Conditions reflect scenarios varying the amount and spatial distribution of conservation investments targeted at waterfowl habitat. With the exception of including winter wheat in crop rotations (which is limited to areas with spring-seeded wheat), we varied conservation amounts throughout the 46 target landscapes. Each scenario was treated as an instantaneous realization of habitat conditions in the Canadian Prairies that remained constant during the study period and provided the basis for estimating reproduction.

Although the land-cover map provides a comprehensive snapshot of land cover, it does not explicitly identify habitats modified by conservation activities. For simulating alternative scenarios, we made assumptions about habitat composition in each landscape and then manipulated habitat proportions to accommodate specific scenarios. For example, we increased the proportion of pasture and decreased the proportion of spring-seeded cropland by equal amounts to simulate the conversion of cropland to grassland. Sufficient data on land cover and conservation activities throughout the study period were not available for the U.S. portion of the PPR. We therefore assumed that reproduction in the U.S. portion paralleled that of the Canadian Prairies throughout the scenarios.

Two alternatives to Observed Conditions represented changes across all conservation areas. To create the No Conservation scenario (Table 1), we altered landscapes in the 2016 land-cover map to reflect a hypothetical lack of habitat conservation over the 10-year period. Specifically, we reverted all restored or protected nesting habitat (i.e., grasslands, wetlands, and winter wheat) to spring-seeded cropland to represent a landscape before observed conservation activities were applied. The number of pintails per landscape were then reduced based on the area of restored wetlands lost using models developed by Bartzen^121^. We generated the Increase Conservation scenario by doubling the acreage per conserved habitat type except winter wheat, which was held at the same acreage as for Observed Conditions.

Because of its importance to nesting pintails, we developed three scenarios focused on winter wheat (Table 1). Under No Winter Wheat, all winter wheat on the 2016 land cover map was reverted to spring-seeded cropland to reflect expected conditions in the absence of funding for winter wheat. The Increase Winter Wheat scenario reflects the stated goal of the Prairie Habitat Joint Venture^51^, to have 30% of the spring-seeded wheat acreage in the Canadian Prairies converted to rotations with winter wheat. We also examined an Increase Winter Wheat - Targeted scenario. Here, we modified the previous scenario by only applying winter-wheat rotations in landscapes with >2.3 pintail pairs km^−2^ (>6 pairs mi^−2^) according to estimated population densities predicted from the species distribution model.

In addition to these a priori scenarios, we conducted a post hoc analysis to determine the amount of spring-seeded cropland that would need to be converted to idle grassland in order to achieve specific population sizes at the continental scale. In particular, we adjusted the amount converted while assuming that winter wheat was included in 23 K km^2^ of crop rotation congruent with the Increase Winter Wheat scenario (Table 1).

Cost estimates for each scenario were based on financial data provided by DUC. Total costs incurred over the 10-year study period were itemized according to the main types of habitat conservation: conversion of spring-seeded cropland to hayland/pasture or idle grassland; increasing amount of cropland with winter wheat in the rotation; protection of existing grasslands and wetlands; and restoration of wetlands.

Along with scenarios for habitat conservation, we considered a range of harvest rates according to existing strategies for harvesting pintails along with the associated uncertainty about actual harvest levels resulting from these strategies. The first strategy assumes a policy of no harvest and closed seasons for hunting pintails. The second assumes a daily bag limit of one pintail. To estimate the range of harvest rates expected under the latter policy, we used annual harvest rates^122^ for years when the daily bag limit for pintails was set to one (i.e., 1988–1996 and 1998–2008) based on yearly rulings in the federal registry e.g.^123^. We fitted a generalized linear mixed model to these harvest data, using a logit link function and year as a random intercept. We determined the baseline (i.e., for adult females) harvest rates that respectively yielded the simulated total harvest rates matching the empirically estimated harvest rate. This baseline harvest rate was used as an input to the population model, which accounts for differential vulnerabilities among age and sex cohorts and crippling loss^19^.

### Population model structure and parameters

We examined effects of changes in age ratio, as mediated by scenarios for harvest and habitat conservation, on equilibrium population size of pintails across their core breeding and wintering regions in North America (Fig. 4). We now briefly summarize the structure of the population model, as the details are described elsewhere^19^. The population model includes two age classes, both sexes, and the five core regions. Core breeding regions include the entire PPR (including portions in Canada and in the U.S.), Alaska, and the northern unsurveyed area. The latter region includes low-quality habitats within the PPR and in northern boreal portions of Canada beyond the PPR. Although this area spatially overlaps the PPR, it is modeled as a separate region to reflect important characteristics of the species distribution and behavior. The western wintering region includes the Pacific Flyway states of California and Oregon, and the central breeding region includes the Central Flyway states of Texas and Louisiana. We updated the structure and parameters of the original model to match current understanding about pintail population dynamics.

First, we changed the initial population size at the start of breeding from 3.2 M to 4.0 M as the sum across all three regions. The latter value is the long-term average of the breeding population based on annual surveys (1955–2014), which is the NAWMP^46^ population goal for pintails. We then removed density-dependent reproduction within breeding regions. The only evidence for density dependence on the breeding grounds is at the continental scale^19^, and regional-scale density dependence has not been detected during long-term studies within the PPR^112^ nor within Alaska (P. L. Flint 2010, oral communication). This modification renders the model with two density-dependent mechanisms. One occurs during spring migration when an increasing proportion of adults settle in poor-quality breeding habitat as abundance increases. The other density-dependent process in the model is the reduction of post-harvest survival with increasing abundance on the wintering grounds^19^.

The estimated age ratio (computed from the reproduction model) for the Observed Conditions scenario was significantly lower than the one used in the original population model. We therefore rescaled age ratios for the remaining breeding regions based on our new estimate for PPR from the Observed Conditions scenario. In particular, we multiplied the original age ratio ${R}_{i}$ by ${R}_{PPR}^{{\prime} }/{R}_{PPR}$, where *i* indexes the other two breeding regions, ${R}_{PPR}^{\text{'}}\,$is the estimate of age ratio for PPR based on the Observed Conditions scenario in the current study, and ${R}_{PPR}$ is the baseline estimate used in the original population model. This calculation is based on the assumption that age ratios in the original model were being overestimated by the same percentage across regions.

Last, we modified several survival rates. We computed a breeding-season survival rate for males of 0.96 (Agresti–Coull 95% confidence interval: 0.89, 0.99)^124,125^, which was based on 76 of 79 radio-marked pintails who survived from early April through early July in landscapes within southern portions of Manitoba and Saskatchewan during 1998 and 1999^126^. This is lower than the original estimate, which was only based on paired males. We also reduced natural mortality rate during fall (i.e., from the start of fall migration to the end of the hunting season) for all cohorts by 0.04. This reduction in fall mortality leads to an annual female survival rate of 0.67, which matches the estimate for the Western and Central Flyways^32^. The modified population model allowed us to estimate equilibrium abundance at the continental scale under each scenario.

### Analysis

For each scenario, we estimated the following parameters within the PPR: conservation cost, habitat availability, hen success, and age ratio at the end of the breeding season. At the continental scale, we projected equilibrium population size at the start of breeding across all three core breeding regions as a function of age ratios. The set of age ratios was based on the modeled estimate for each of the a priori scenarios for habitat conservation along with those predicted under the post hoc scenarios.

Although the model is deterministic, we ran each scenario using the mean and 95% confidence limits for age ratio, male survival during breeding, and harvest rate. We held the remaining parameters constant, because empirical data for computing confidence intervals for these are lacking. To estimate standard error for age ratio, we integrated uncertainties about clutch size, hen success, duckling survival, and female breeding season survival within the reproduction model by using the delta method^127^. We then multiplied this standard error by 1.96 to obtain the upper and lower prediction intervals for age ratio under each habitat conservation scenario. To model the 95% confidence interval for harvest rate for all individuals under a daily bag limit of one, we extracted the 2.5^th^ and 97.5^th^ quantiles from 1000 bootstrap iterations of the mixed logistic regression using the bootMer function in program R^128^. The method for estimating the confidence interval breeding survival of adult males is given under “Population model structure and parameters”, above.

The analysis was conducted using SAS version 9.4 (SAS Institute, Cary, North Carolina, USA8) for the reproduction model and program R^128^ for the remaining parameters and population model. All monetary values are given in 2016 USD.

## Supplementary information

Supplementary material.

## Data Availability

The data and code used for the analysis in this study are available from the corresponding author upon request.
