# Publisher Correction: Linking landscape-scale conservation to regional and continental outcomes for a migratory species

**DOI:** 10.1038/s41598-021-91551-2

**Published:** 2021-06-01

**Authors:** B. J. Mattsson, J. H. Devries, J. A. Dubovsky, D. Semmens, W. E. Thogmartin, J. J. Derbridge, L. Lopez-Hoffman

**Affiliations:** 1 Institute of Wildlife Biology and Game Management, University of Natural Resources and Life Sciences, 1180 Vienna, Austria; 2 Ducks Unlimited Canada, Stonewall, MB R0C2Z0 Canada; 3 Division of Migratory Bird Management, U.S. Fish and Wildlife Service, Lakewood, CO 80215 USA; 4 Geosciences and Environmental Change Science Center, U.S. Geological Survey, Denver, CO 80225 USA; 5 Upper Midwest Environmental Sciences Center, U.S. Geological Survey, La Crosse, WI 54603 USA; 6 School of Natural Resources and Environment, The University of Arizona, Tucson, AZ 85719 USA; 7 Udall Center for Studies in Public Policy, The University of Arizona, Tucson, AZ 85719 USA

Correction to: *Scientific Reports* 10.1038/s41598-020-61058-3, published online 18 March 2020

The original version of this Article contained an error in Figure 3 where the *h* values were omitted. The original Figure 3 and accompanying legend appear below.

**Figure 3:**
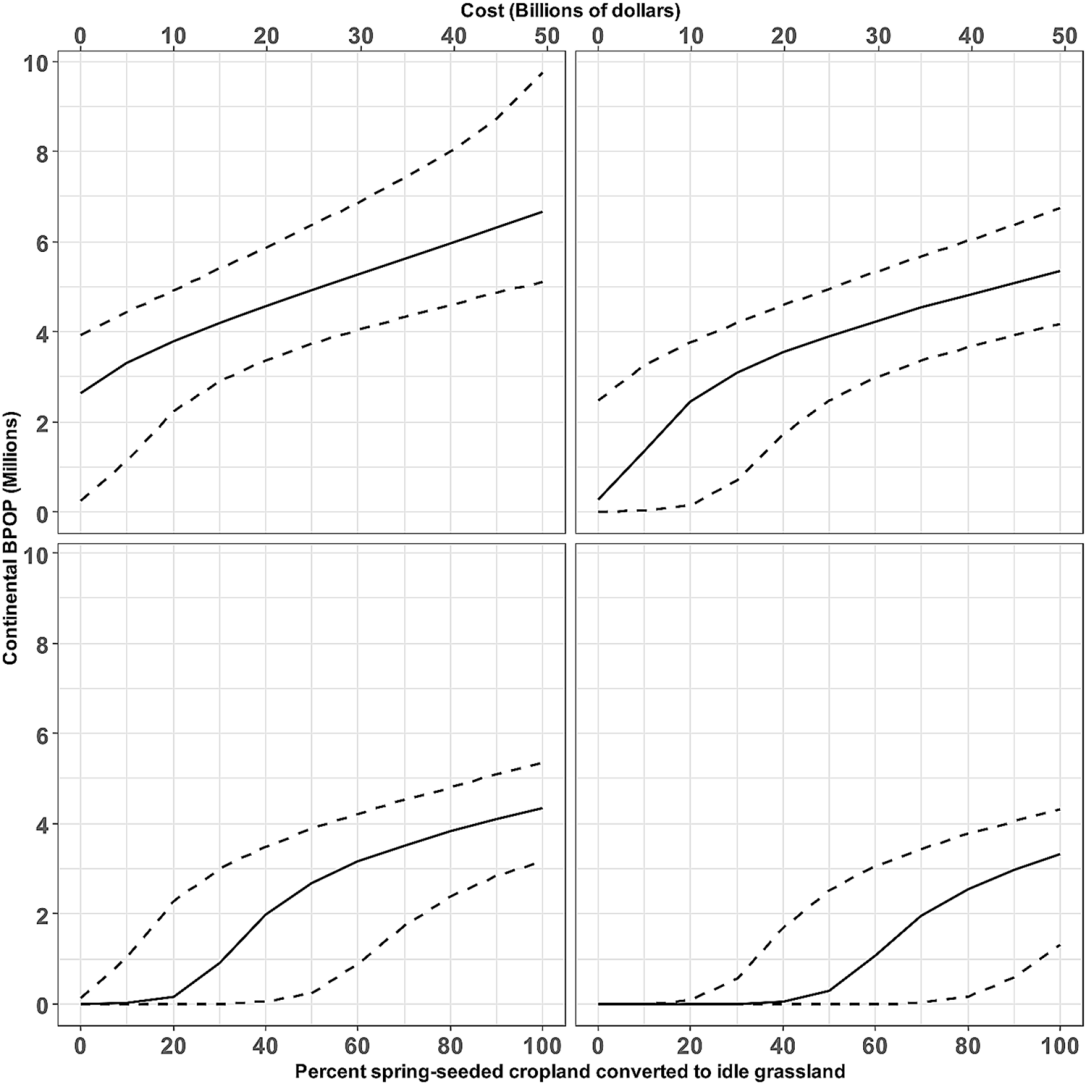
Predicted relationship between conversion of spring-seeded cropland to idle grassland within the Prairie Pothole Region and the equilibrium population size of northern pintails at the start of the breeding season. The population size at the x-intercept (i.e., % converted = 0) corresponds with the Increase Winter Wheat scenario. Panels represent predicted relationships under particular proportions of all pintails harvested (i.e., harvest rate; *h*). A harvest rate of zero represents a closed hunting season, and the remaining represent the uncertainty about harvest rate expected under a bag limit of one pintail. Cost of habitat conversion (secondary x-axis) is given in 2016 USD. Solid line is based on mean values for all parameters, and the dashed lines are based on the upper and lower 95% confidence intervals for parameters derived from available empirical data.

The original Article has been corrected.

