# Supplementary material for: Linking landscape-scale conservation to regional and continental outcomes for a migratory species
Source: Sci Rep. 2020 Mar 18;10:4968. doi: 10.1038/s41598-020-61058-3 (PMC7080806; doi:10.1038/s41598-020-61058-3)
Supplement: Supplementary file 1 — Supplementary material. [file 41598_2020_61058_MOESM1_ESM.docx]

**Title:** Linking landscape-scale conservation to regional and continental outcomes for a migratory species

**Authors**: B.J. Mattsson**^[[1]](#footnote-1)^**^*^, J.H. Devries**^[[2]](#footnote-2)^**, J.A. Dubovsky**^[[3]](#footnote-3)^**, D. Semmens^[[4]](#footnote-4)^, W.E. Thogmartin^[[5]](#footnote-5)^, J.J. Derbridge^[[6]](#footnote-6)^, and L. Lopez-Hoffman^6,^^[[7]](#footnote-7)^


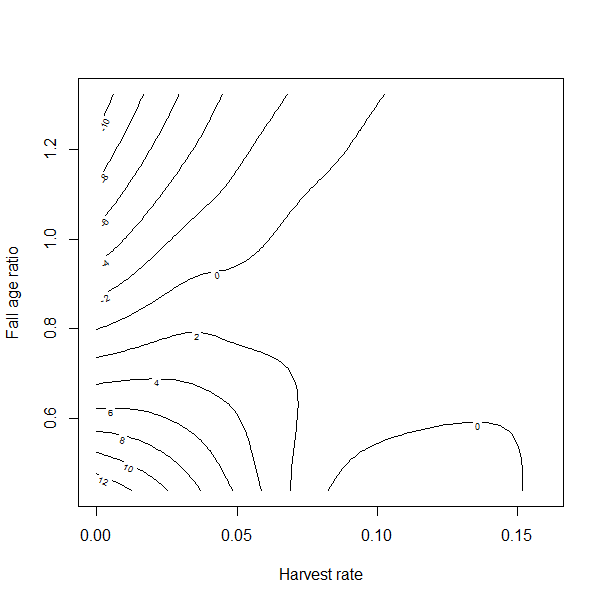

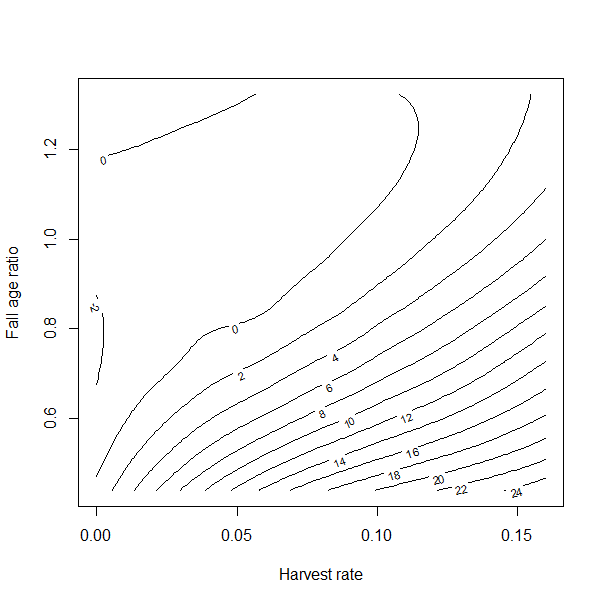


Baseline harvest rate

Fig. S1. Actual (in thousands; A) and proportional (B) differences when subtracting lower from upper confidence limits of predicted population size for northern pintails in North America when varying male breeding survival from 0.89 to 0.99. Fall age ratios varied as a function of habitat conservation scenarios in the Prairie Pothole region, and baseline harvest rates represent a daily bag limit of one while accounting for uncertainty.


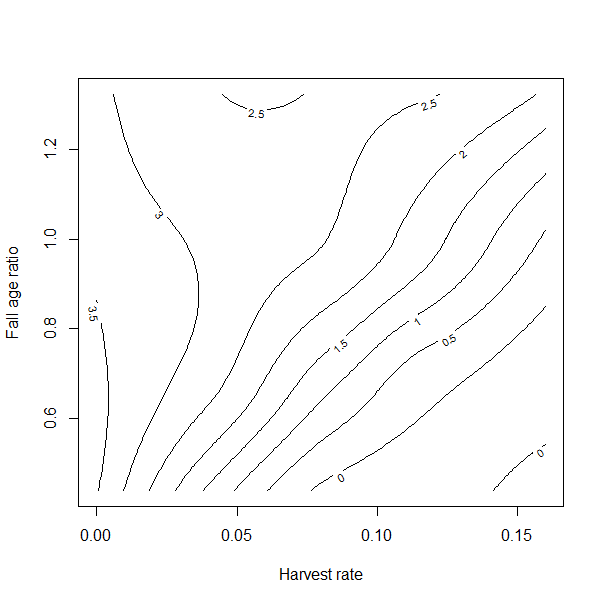

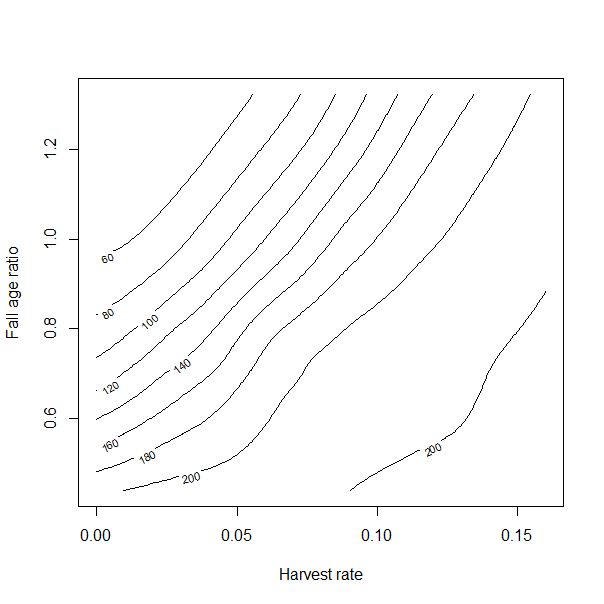


Baseline harvest rate

Fig. S2. Actual (in millions; A) and percent (B) differences when subtracting lower from upper confidence limits of predicted population size for northern pintails in North America when varying fall age ratio in the Prairie Pothole Region between its 95% confidence intervals across a range of averages (x-axis). The mean age ratios varied as a function of habitat conservation scenarios in the Prairie Pothole region, and baseline harvest rates represent a daily bag limit of one while accounting for uncertainty.

1. Institute of Wildlife Biology and Game Management, University of Natural Resources and Life Sciences, Vienna 1180, Austria. *Corresponding author e-mail address: [brady.mattsson@boku.ac.at](mailto:brady.mattsson@boku.ac.at) [↑](#footnote-ref-1)
2. Ducks Unlimited Canada, Stonewall, MB R0C2Z0, Canada. [↑](#footnote-ref-2)
3. Division of Migratory Bird Management, U.S. Fish and Wildlife Service, Lakewood, CO 80215, USA. [↑](#footnote-ref-3)
4. Geosciences and Environmental Change Science Center, U.S. Geological Survey, Denver, CO 80225, USA [↑](#footnote-ref-4)
5. Upper Midwest Environmental Sciences Center, U.S. Geological Survey, La Crosse, WI 54603, USA [↑](#footnote-ref-5)
6. School of Natural Resources and Environment, The University of Arizona, Tucson, AZ 85719, USA [↑](#footnote-ref-6)
7. Udall Center for Studies in Public Policy, The University of Arizona, Tucson, AZ 85719, USA [↑](#footnote-ref-7)
